# Non-modifiable Factors of Coronary Artery Stenosis in Late Onset Patients with Coronary Artery Disease in Southern Iranian Population

**DOI:** 10.5681/jcvtr.2014.010

**Published:** 2014-03-21

**Authors:** Seyeed Mohammad Bager Tabei, Sara Senemar, Babak Saffari, Zeinab Ahmadi, Somayeh Haqparast

**Affiliations:** ^1^Department of Medical Genetics, Shiraz University of Medical Sciences, Shiraz, Iran; ^2^Department of Medical Genetics, Shiraz University of Medical Sciences, Shiraz, Iran; ^3^Department of Biology, Tehran University of Medical Sciences, Tehran , Iran

**Keywords:** Premature, Risk Factors, Coronary Artery Disease Familial, Myocardial Infarction, Consanguinity

## Abstract

***Introduction:*** Coronary Artery disease (CAD) is influenced by genetic factors, environment and culture behavior. The aim of the present study was to evaluate some non-modifiable risk factors of coronary heart disease such as sex, age, family history and consanguineous marriage.

***Methods:*** This is a case-control study. The study population consisted of 200 fifteen or more years old. Data were collected on 200 patients with positive angiography and 200 control subjects with negative angiography. Positive angiography was defined as coronary diameter cut greater than 50%. Statistical analysis was conducted using SPSS 11.5. In this study, data were collected through a checklist. Logistic regression and stratification were used to determine the impact of age, gender, family history, and consanguinity on the risk of stenosis.

***Results:*** The percentage of men in patients and controls were 89% and 29%, respectively. As to gender, a significant association was found between patients and controls of CAD (CI 95%, 4.014-10.052, OR 6.352). Gender was determined as a risk factor for CAD. Family history of myocardial infarction did not show a significant effect on the artery stenosis. As to consanguinity of the parents, there was no significant association between patients and controls of CAD (P> 0.05).

***Conclusion:*** These researches show that ageing increases the risk of coronary heart stenosis; also, females are more than men protected against this disease. The impact of family history of myocardial infarction and consanguineous marriage were not associated with of CAD.

## Introduction

The main cause of death among men and women is the cardiovascular disease.^1^ According to WHO estimates, annually the cause of 16.7 million deaths around the world is cardiovascular diseases.^2^ Coronary artery diseases (CAD) have been estimated to be the leading cause of death in developing countries in 2010.^3^ The lowest age-related mortality rates are in the advanced industrialized countries and parts of Latin America, whereas currently the highest rates are found in Eastern Europe and a few low and middle income countries. Overall, age-adjusted cardiovascular disease death rates are at present higher in major low and middle-income countries than in developed countries.^4,5^ Various forms of cardiovascular disease include hypertension, atherosclerosis, coronary heart attack, heart failure, stroke and other vascular diseases.^1^ Some risk factors of coronary heart diseases are modifiable, while others are not controllable. The chance of coronary artery disease is raised in the face of higher risk factors. Globally, high blood pressure, smoking, elevated blood glucose, physical inactivity, overweight and obesity are the six leading risk factors. In capitalist countries and developed countries, alcohol ranks in the top of risk factors of coronary heart disease, but in developed countries the top risk factor are lack of safe water, unsafe sex, and under nutrition.^6^ Risk factors which are modifiable or treatable such as high blood pressure, high blood lipids, and high blood glucose are of biological nature, and have a wide impact on the global incidence and burden of the disease^7^ The major non-adjustment risk factors that we discussed in this research are gender, age, family history, consanguinity marriage.

### Gender

Sex is considered as an untreatable and non-modifiable risk factor in that women with more than 55 years of age are more susceptible to disease than men. Women before menopause are protected against CAD, because of the impact of sex hormones function.^8^

### Age

Risk of CAD increases with age. Men of younger age are more vulnerable to attacks than women of the same age.^9^ The risk of cardiovascular disease begins to rise in postmenopausal females.^10^

### Family history

Premature coronary vascular disease occurs in families with the history of CAD. The disease can be prevented by identifying the families with positive history of CAD, especially in people who have parents or siblings with artery disease or death in the family because of a history of CAD at an early age.^1,2,11^ Slack et al. proved that the risk of getting coronary heart disease among first-degree relatives of patients with CHD was 2.5 to 7 times higher than the relatives of the controls.^12^

### Consanguineous marriage

This is important in cardiovascular disease. The frequency of consanguineous marriage is common in the third world countries which are 50.3%, 54.3%, 28.9%, 38.6%, and 25% in Jordan, Kuwait, Egypt, Iran, and Lebanon, respectively.^13^ Genetic studies lead to recognition of new complex disease causing variants, and focus on the association between polymorphism and risk of coronary artery disease .^14^

The present study was designed to analyze and compare non-modifiable risk factors in patients with PCAD and in those with late -onset CAD.

## Materials and methods

This is a case-control study and our sampling method was convenient. We selected 400 subjects (patients and controls) from cardiac catheterization laboratory that underwent diagnostic coronary angiography and positive angiography was defined as coronary diameter cut greater than 50%. They were selected based on the severity of the vessel stenosis and age. The data on variables were collected by a checklist. The study population included 200 patients (aged 51-81; mean age 60±7.4) with positive angiography and over 50 years of age. The control group (aged 51-82; mean age 58.25±7.65) had negative atherosclerosis. The checklist was designed by the researcher considering sex, age of the onset of the disease, family history and consanguinity of parents. Statistical analysis was conducted using the statistical package for social science SPSS (version 11.5). Two-sided P value≤ 0.05 was considered statistically significant for all the analyses. Logistic regression was used to determine the impact of all variables (age, gender, family history and consanguinity) on the risk of coronary stenosis of CAD.

## Results

Genders are different among patient and control groups (percents of male patients in our sample is 59.9% and female patients is 66.6% and the percent male controls is 53.3%, in female controls 64.9% with late onset coronary artery disease ( Table 1). There was a statistically a significant association between gender and late onset age referred to community health center (*P*<0.05) ( Tables 2 and 3).

**Table 1:** Conventional risk factors in & CAD patient and control subjects CAD (n= 400)

**Parameter**	**Patients (n=200) **	**Controls (n=200)**	***P***
** Age[yr], (mean)±SD **	60±7.4	58.25±7.65	>0.05
** Sex (male/female) **	88/112	105/95	<0.001
**Familial History (n%)**	47.5 (95)	53 (106)	>0.05
**Parents Consanguinity Marriage (n%)**	46.5(93)	42 (84)	>0.05

**Table 2:** Mean age in subjects with Late Coronary Artery Disease

**Study population**	**Sex(M:F)**	**Mean** **age± SD**	***P***
**Patients**	Male	59.9 ±9.1	0.04
	Female	66.6±9.22	
**Control**	Male	53.3± 3.47	<0.001
	Female	64.9 ± 9.97	

**Table 3:** Association between Sex and Coronary heart disease

	**Gender**	**β coefficient**	**Standard error**	**OR**	**95% CI**	***P***
**CAD**	Male/Female	1.849	0.234	6.352	4.014-10.052	<0.001

Based on the comparison of the mean age among women and men, the result showed that mean age of patient and control groups was higher in women than men. The result revealed that there were significant age differences between men and women in both groups ( Table 2).

Family history of myocardial infarction (MI) was not significant in case and control groups of CAD (*P* value= 0.430). The percentages of myocardial infarction in late onset coronary heart disease are 47.5% in patients and 53% in the control group, as shown in Tables 1 and 4. There was no significant relationship between the degree of consanguinity in parents of CAD ( Table 1) The percentages of consanguinity in parents are 46.5% in patients and in 42% in the control group, as shown in Table1  (P value= 0.625). Differences in consanguinity degree of parents regarding CAD patients and controls are displayed in Table 5.

**Table 4:** Percentage of myocardial infractions in late onset coronary heart disease

** History of MI degree with a ** **history MI**	**Percentage of History of** ** MI(N) **	**Percent age of relative' s MI**		
		** 1°**	**2°**	**3°**
**MI Controls**	53(106)	81.5	12	3
**MI** **Cases**	47.5(95)	82	15	2

We removed the number of cases with a family history higher than Grade 3

**Table 5:** Consanguinity degree of parents in CAD groups

**Groups**	**Parents** ** consanguinity**		**Percent of ** ** Parents consanguinity (N) **	**Percent of relationship consanguinity**		
				**2°**	**3°**	**4°**
**Patients**	CAD	No relative	53.5(107)	-	-	-
		Relative	46.5(93)	6	25	5
**Controls**	CAD	No relative	58(116)	-	-	-
		Relative	42(84)	4	18	9

We removed the number of cases with a family history higher than Grade 3

## Discussion

Coronary artery disease is a multifactorial disease in which the response to different environmental and genetic influences is important. Different genetic polymorphisms, marriage, kinship and family history, age and gender are unchangeable adjustment factors in human life. In this study, we analyzed some non-modifiable factors of CAD.

The first non-modifiable factor is age. This study showed that the age factor of patients and controls was matched (P-values were not significant). The mean age did not show a statistically significant difference, but it seems that this disease occurs in the elderly. When we compared the previous reports in Iran with our results, it was found that older adults with coronary atherosclerosis living in Fars province had the same mean ages as those residing in other provinces in Iran.^15^ Also, the present results may partly confirm the theory that ageing raises the risk of disease. As age creeps on, the risk of coronary atherosclerosis and vein problems increases.

The second irreversible risk factor is sex differences in cardiovascular disease. Data from the present study showed the number of males who suffered from coronary artery disease was higher in CAD group. This indicates that among people referred to the community health center with symptoms of chest pain and diagnosed with angiography, the percentage of women with risk of coronary atherosclerosis is fewer than men. More advanced stenosis show in over 55 years old women compared with men in the same age.^16,17^ There is a possible explanation for gender differences. The genetic factor like changes in lipid profile, obesity, hypertension, glucose intolerance, diabetes mellitus type II and environmental factors such as anger, aggression, addiction, and severe economic problems of our society account for differences in the percentage of gender in developing CAD. On the other hand the protective action of sex hormones

(Estrogen and progesterone) has been observed in young women. In our research, In Iranian females, it is about 6 times more than men. Perhaps this occurred because it removes the protective effects of these hormones due to menopause (because of increasing age). Different studies show hormonal changes in the protective mechanism of the indirect effects of fat, altered metabolism (glycoid) and direct effects on vascular work.^18,19^ Before menopause, the risk of cardiovascular disease in woman is much less than men, mostly due to protective effect of the female hormone estrogen. Although the effects of estrogen are not fully understood, it appears that it has a positive effect on blood cholesterol levels in young women. The onset of menopause, when estrogen production stops, the protective effect of estrogen is lost. Risk of cardiovascular disease in postmenopausal women who are under hormone replacement therapy reduces gradually. To relieve symptoms of menopause, e.g. hot flashes, hormone therapy which also protects them against osteoporosis is used. HRT may increase the risk of breast cancer and some other cancers, but the benefits of hormone therapy in most women are much more than its disadvantages.

Another important independent risk factor for CAD is family history of MI. Other studies have revealed that having one or more first-degree relatives with CAD are associated with a three-to six fold increase in the risk .^20,21^ In this study, there was no significant association between subjects with family history of MI and risk of coronary artery disease in CAD although in other studies a significant association between CAD and family history had been observed.^22-24^ Much of the familial aggregation of CAD might be explained by the familial aggregation of established risk factors, such as high LDL cholesterol, low HDL cholesterol, and diabetes.^25,26^ But the interactions of the genetic, environmental, cultural, and behavioral risk factors shared by family members may be too intricate to assess with usual statistical methods. The results obtained from our data are different

from those of other studies because the percentage of myocardial infarction is high in our population and this disease is the first factor of mortality . Also it is stated that hereditary and genetic factors and gene resource of Iranian population affect the heart disease less than the environmental factors. 81.5% and 82% of the first- degree relatives with a family history of CAD in the control and patient groups were afflicted with the disease, respectively. They are the highest level recorded in the world because MI is extremely common in Iranian population and this is the first cause of mortality in our country.^20,27,28^ However, our results confirm the findings of previous studies.^29^

In the present study, the results showed that there was no significant difference between coronary heart disease and consanguinity of parents. But the prevalence of third degree consanguinity in cases and controls of CAD (despite of no significant differences among case and control) was high. The reason of this consequence is the high percentage of third degree consanguinity in Iranian population.^30^

In conclusion, this study showed that in patients with late CAD Coronary risk increases with ageing and the incidence of the disease was found to be more frequent in female patients than in male counterparts in the same age range. The study added that subjects who had more family history of MI or consanguinity marriage did not significantly the risk of coronary artery disease in PCAD and CAD groups.

Also, I suggest that future studies be conducted on larger populations, other parts of the country other provinces in Iran and more health centers.

## Acknowledgements

Support of this study by Fars Province Branch of Iranian Academic Center for Education, Culture & Research (ACECR) is acknowledged. The authors wish to thank all the participants of this study, Dr. MB Sharifkazemi for kindly providing the clinical samples and nursing staff of Saadi, Nemazee and Kowsar hospitals for their valuable cooperation. The authors would like to thank Dr. Nasrin Shokrpour for editorial assistance and Mrs Sareh Roosta for statistical analysis at Center for Development of Clinical Research of Nemazee Hospital.

## Ethical issues

All patients gave written informed consents and the study

was approved by our local Ethics Committee.

## Competing interests

The authors declare that they have no competing interests.
